# Hemorrhage complications in obstetric antiphospholipid syndrome: Risk factors and association with adverse pregnancy outcomes

**DOI:** 10.3389/fimmu.2023.1145146

**Published:** 2023-03-17

**Authors:** Yongjing Luo, Jiayang Jin, Yani Yan, Mengyao Zhang, Lei Hou, Yuke Hou, Qiuyan Pei, Chun Li

**Affiliations:** ^1^ Department of Rheumatology and Immunology, Peking University People’s Hospital, Beijing, China; ^2^ Beijing Key Laboratory for Rheumatism Mechanism and Immune Diagnosis (BZ0135), Beijing, China; ^3^ Obstetrics and Gynecology Department, Peking University People’s Hospital, Beijing, China; ^4^ Department of Rheumatology and Immunology, The Second Affiliated Hospital of Guizhou University of Traditional Chinese Medicine, Guiyang, China

**Keywords:** antiphospholipid syndrome, hemorrhage complications, mucocutaneous hemorrhage, preterm delivery prior to 34 weeks, adverse pregnancy outcomes

## Abstract

**Background:**

Bleeding complications are recognized as relatively infrequent manifestations of antiphospholipid syndrome (APS), and the safety of antithrombotic therapy during pregnancy is of concern. This study aims to assess the risk factors and possible associations between bleeding complications and adverse pregnancy outcomes (APOs) in patients with APS.

**Methods:**

A retrospective cohort study was conducted at the Peking University People’s Hospital. The clinical and immunologic features, bleeding complications, treatment, and pregnancy outcomes of patients with APS were collected. Univariate and multivariate logistic regression analyses were applied to assess the associations between APOs and bleeding complications.

**Results:**

A total of 176 participants with obstetric APS were included in the analysis. There were 66 (37.50%) patients with APS with hemorrhage complications and 86 (48.86%) patients with APS with APOs. Mucocutaneous hemorrhage was associated with APOs including fetal death after 12 weeks [odds ratio (OR) = 10.73, 95% confidence interval (CI): 1.61–71.74, p = 0.014], preterm delivery prior to 34 weeks (OR = 8.30, 95% CI: 2.31–29.84, p = 0.001), and small for gestational age (OR = 4.17, 95% CI: 1.22–14.21, p = 0.023) in univariate logistic regression analyses. It also independently associated with preterm delivery prior to 34 weeks (OR = 40.29, 95% CI: 1.45–1121.32, p = 0.030) in multivariate logistic regression analyses. Receiver operating characteristic (ROC) analysis evaluating the accuracy of these factors for preterm delivery prior to 34 weeks showed that the area under ROC curve was 0.871.

**Conclusion:**

The study shows that mucocutaneous hemorrhage may be an indication of the occurrence of APOs in obstetric patients with APS.

## Introduction

Antiphospholipid syndrome (APS) is an autoimmune disease characterized by recurrent miscarriage and/or thrombosis and the presence of high titers of antiphospholipid antibodies (aPLs) (1–4). The aPLs include anticardiolipin antibodies (aCLs), anti-β2 glycoprotein I antibodies (aβ2-GPIs), and lupus anticoagulant (LA). It has been reported that women with APS are at increased risk of adverse pregnancy outcomes (APOs), including preeclampsia, pregnancy loss, preterm delivery, and perinatal mortality (1, 5, 6). Whether different clinical manifestations and immune indicators are associated with an increased risk of APOs is still controversial. Several clinical studies have revealed risk factors for APOs, including multiple previous pregnancy losses (7), previous thrombosis (8, 9), high titers of aPLs (9–12), and large number of autoantibodies (8, 9, 13, 14). Patients with triple aPL positivity and prior thrombosis were found to be at highest risk for obstetric complications (15).

Women who fulfill the Sydney criteria and have had no previous thrombotic events are identified as patients with obstetric APS (OAPS) (16). Thrombosis and inflammation are involved in the pathophysiological changes of OAPS. aPLs lead to placental dysfunction by reducing the proliferation and invasion of extravillous trophoblasts and by triggering inflammation at the maternal-fetal interface, which eventually leads to APOs (4). The associated inflammatory responses, especially complement activation, recruitment and stimulation of neutrophils (17), overexpression of tissue factor in monocytes (18), and dysregulation of the angiogenic factors required for normal placental development (18), are the major causes of placental insufficiency, fetal loss, and growth restriction. The management of APS has been controversial, and anticoagulant therapy is most commonly used treatment (4). Considering the effect of thrombotic and inflammatory factors in the pathophysiology of OAPS, low–molecular weight heparin (LMWH) and low-dose acetylsalicylic acid (LDASA) are commonly recommended for the management of patients with APS during pregnancy (19), and the specific treatment in clinical practice needs to be determined according to the maternal and fetal conditions. It has been shown that heparin prevents pregnancy complications in patients with OAPS mainly by blocking complement activation rather than just functioning in anticoagulation (17). However, the application of antithrombotic therapy may increase the risk of bleeding, leading to the occurrence of bleeding complications, such as mucocutaneous hemorrhage, vaginal bleeding, subchorionic hemorrhage, and postpartum hemorrhage. In addition, capillaritis, microthrombosis, antiprothrombin antibodies, and thrombocytopenia may also increase the risk of bleeding in patients (20).

Although studies have evaluated the risk factors for APOs, there are few cases of bleeding complications in patients with OAPS reported. Therefore, the aim of this study is to assess the association between bleeding complications and APOs in patients with OAPS and to determine whether different antithrombotic therapies increase the risk of bleeding.

## Methods

### Patients

Data for the retrospective cohort study were collected at the Peking University People’s Hospital. Data from all patients with OAPS were retrospectively reviewed between January 2015 and June 2021. All patients fulfilled the Sydney classification criteria of APS (1). The inclusion criteria were 1) live fetus diagnosed by ultrasonic examination at 6 weeks, 2) diagnosis of primary APS before or during pregnancy, and 3) complete clinical and follow-up data. The exclusion criteria were 1) voluntary interruption of pregnancy and 2) missing clinical data or follow-up data. This study was conducted in accordance with the Declaration of Helsinki and was approved by the ethics committee of the Peking University People’s Hospital (2019PHB252). All patients signed written informed consents before participating in this study.

The following data were collected: 1) demographic and gestational characteristics: age, age of disease onset, primipara, APS diagnosis before pregnancy, gestational week, previous abortion history, use of assisted reproduction techniques, and history of cesarean section; 2) clinical features: history of thrombosis and thrombocytopenia; serology data, namely, antinuclear antibody (ANA), hypocomplementemia, high titers of aβ2-GPIs or aCLs, double aPL positivity, and triple aPL positivity; treatment, namely, prednisone (Pred), hydroxychloroquine (HCQ), LMWH, LDASA, intravenous immunoglobulin (IVIG), and LMWH + LDASA; bleeding complications, namely, vaginal bleeding, subchorionic hemorrhage, mucocutaneous hemorrhage, and postpartum hemorrhage; abnormal umbilical blood flow, and abnormal middle cerebral artery blood flow; 3) APOs of patients with APS: fetal death after 12 weeks of gestation, preterm delivery prior to 34 weeks, preeclampsia, placental insufficiency, small for gestational age (SGA), fetal death, preterm delivery prior to 37 weeks, and fetal distress (21).

### Bleeding complications

The symptoms and signs of the patients all were taken from the medical record. Vaginal bleeding refers to the outflow of blood from the uterus during pregnancy. Subchorionic hemorrhage was defined as dark areas of liquid that can be seen around the chorion in the uterine cavity during ultrasound examination. Mucocutaneous hemorrhage comprised petechiae (diameter of no more than 2 mm), purpura (diameter between 3 and 5 mm), ecchymosis (subcutaneous flaky hemorrhage with a diameter > 5 mm), subcutaneous and deep tissue hematoma (massive subcutaneous hemorrhage and ecchymosis with obvious bulging and swelling of the skin or joint cavity), blood blisters (dark black or purplish red blister-like hemorrhages of different sizes, which are mostly seen in the mouth and tongue), epistaxis, and gingival bleeding. Postpartum hemorrhage in China is defined as loss of more than 500 ml of blood within 24 h after delivery. Postpartum hemorrhage was measured using visual inspection, the area method, the weighing method, the shock index method, and hemoglobin determination.

### Adverse pregnancy outcomes

The signs of placental insufficiency comprise Oligohydramnios, fetal growth restriction, the absence of diastolic flow in the umbilical artery, and an abnormal fetal heart rate (22–24). Patients were monitored for these signs during antenatal examination. Preterm delivery was defined as spontaneous delivery and doctor’s assessment of the need to terminate pregnancy because of gestational hypertension, preeclampsia, placental insufficiency, and SGA children (birth weight < 5th percentile) (25). Patients with preterm delivery prior to 34 weeks were classified as an overall group. Preeclampsia was defined as the presence of elevated blood pressure with positive urine protein or the absence of urine protein, thrombocytopenia, impaired liver and kidney function, pulmonary edema, and new central nervous system abnormalities or visual impairment after 20 weeks. SGA was defined as a child born with birth weight below the fifth percentile for a population with the same gestational age and sex.

### Statistical analysis

Quantitative variables were expressed as the mean ± standard deviation (SD) or the median and the interquartile range (IQR) depending on their distribution, and qualitative variables were expressed as frequencies and percentages. The normality of the distribution was checked by graphical inspection and the Shapiro–Wilk test. The significance of the qualitative variables was assessed using the chi-square test and Fisher’s exact test, and that of quantitative variables was assessed using the Mann–Whitney U-test. The significance of associations between bleeding complications and APOs in patients with APS was first evaluated by univariate logistic analysis. The p-values and odds ratios (ORs) with confidence intervals (CIs) were calculated. Variables with p < 0.05 in univariate logistic analysis were analyzed by multivariate logistic regression analyses. Receiver operating characteristic (ROC) curve and area under the ROC curve (AUC) analyses were used to establish the diagnostic accuracy and the optimal cutoff values. Hierarchical analysis was used to investigate the associations between antithrombotic therapies and APOs in different bleeding complications. The statistical significance level was set at 0.05 for all testing procedures. All analyses were performed with the statistical software R version 3.4.3 (http://www.R-project.org, The R Foundation) and EmpowerStats (http://www.empowerstats.com, X&Y Solutions, Inc., Boston, MA, USA).

## Results

### Characteristics of patients

A total of 176 patients with OAPS were analyzed in the cohort. The average age was 33.20 years, and the average age of disease onset was 31.23 years ± 3.66. The average week of gestation was 37.11 weeks ± 4.06. The incidence of primipara was 73.86%, and 72.73% of patients were diagnosed with APS before pregnancy. In addition to the 174 pure OAPS cases (98.86%), there were two obstetric + thrombotic APS cases (1.14%) and no pure thrombotic APS cases. ANA positivity was noted in 39 (26.35%) cases, and triple aPL positivity was noted in 16 (9.14%) of cases. Regarding treatment, LMWH (87.41%), HCQ (72.73%), and LDASA (69.32%) were the most commonly used therapeutic agents, whereas Pred (31.25%) was used less frequently. Among the 176 patients with OAPS, 86 (48.86%) patients had APOs, among which placental insufficiency (35.80%) and preterm delivery prior to 37 weeks (21.02%) were the most common, followed by SGA (16.48%) and fetal distress (15.91%), and the least common were fetal death after 12 weeks of gestation (2.84%) and fetal death (2.27%). Sixty-six (37.50%) patients had bleeding complications. Vaginal bleeding (20.45%) and subchorionic hemorrhage (11.36%) were the most common complications. Mucocutaneous hemorrhage and postpartum hemorrhage were noted in 12 (6.82%) patients (**Table 1**). The patients in the mucocutaneous hemorrhage group had lower rates of APS diagnosis before pregnancy (41.67% *vs*. 75.00%, p = 0.012) and use of assisted reproduction techniques (0.00% *vs*. 25.00%, p = 0.048) compared with patients in control group (**Supplementary Table 1**). Patients had a higher rate of mucocutaneous hemorrhage in non-antithrombotic treatment group (60.00% *vs*. 1.91%, p < 0.001) (**Supplementary Table 2**).

**Table 1:** Clinical and gestational characteristics of patients with APS.

	Number of patients (Total n = 176)
Age, years	33.20 ± 3.68
Age of disease onset, years	31.23 ± 3.66
Primipara, n (%)	130 (73.86%)
Diagnosed APS before pregnancy, n (%)	128 (72.73%)
Pure obstetrical APS cases, n (%)	174 (98.86%)
Pure thrombotic APS cases, n (%)	0 (0.00%)
Venous, n (%)	0 (0.00%)
Arterial, n (%)	0 (0.00%)
Obstetrical + thrombotic APS cases, n (%)	2 (1.14%)
Thrombosis, n (%)	3 (1.70%)
Thrombocytopenia, n (%)	35 (19.89%)
Serology	
ANA positivity, n (%)	39 (26.35%)
Hypocomplementemia, n (%)	24 (15.00%)
High titers of aβ2-GPIs/aCLs, n (%)	55 (31.43%)
Triple aPL positivity, n (%)	16 (9.14%)
Double aPL positivity, n (%)	40 (22.86%)
**Treatment**	174 (98.86%)
Pred, mg/day	0.00 (0.00–5.00)
Pred, n (%)	55 (31.25%)
HCQ, mg/day	400.00 (0.00–400.00)
HCQ, n (%)	128 (72.73%)
LMWH, IU/day	4100.00 (4000.00–5000.00)
LMWH, n (%)	138 (78.41%)
LDASA, mg/day	75.00 (0.00–85.00)
LDASA, n (%)	122 (69.32%)
LMWH + LDASA, n (%)	98 (55.68%)
IVIG, n (%)	6 (3.41%)
**APOs, n (%)**	86 (48.86%)
Fetal death after 12 weeks of gestation, n (%)	5 (2.84%)
Preterm delivery prior to 34 weeks, n (%)	18 (10.23%)
Preeclampsia, n (%)	23 (13.07%)
Placental insufficiency, n (%)	63 (35.80%)
SGA, n (%)	29 (16.48%)
Fetal death, n (%)	4 (2.27%)
Preterm delivery prior to 37 weeks, n (%)	37 (21.02%)
Fetal distress, n (%)	28 (15.91%)
**Bleeding complications**	66 (37.50%)
Vaginal bleeding, n (%)	36 (20.45%)
Subchorionic hemorrhage, n (%)	20 (11.36%)
Mucocutaneous hemorrhage, n (%)	12 (6.82%)
Postpartum hemorrhage, ml	343.14 ± 259.03
Postpartum hemorrhage, n (%)	12 (6.82%)
Abnormal umbilical blood flow, n (%)	6 (3.41%)
Abnormal middle cerebral artery blood flow, n (%)	3 (1.70%)

APS, antiphospholipid syndrome; Pred, prednisone; HCQ, hydroxychloroquine; LMWH, low–molecular weight heparin; LDASA, low-dose acetylsalicylic acid; IVIG, intravenous immunoglobulin; ANA, antinuclear antibody; aβ2-GPIs, anti-β2 glycoprotein I antibodies; aCLs, anticardiolipin antibodies; aPLs, antiphospholipid antibodies; APOs, adverse pregnancy outcomes; SGA, small for gestational age.

### Univariate logistic regression analyses of the association of bleeding complications with APOs

Mucocutaneous hemorrhage was associated with APOs including fetal death after 12 weeks (OR = 10.73, 95% CI: 1.61–71.74, p = 0.014), preterm delivery prior to 34 weeks (OR = 8.30, 95% CI: 2.31–29.84, p = 0.001), SGA (OR = 4.17, 95% CI: 1.22–14.21, p = 0.023), and preterm delivery prior to 37 weeks (OR = 4.29, 95% CI: 1.30–14.20, p = 0.017) in univariate logistic regression analyses. However, vaginal bleeding, subchorionic hemorrhage, and postpartum hemorrhage were not associated with APOs (p > 0.05) (**Table 2**).

**Table 2:** Univariate logistic regression analyses of the bleeding complications associated with APOs in patients with APS.

	Bleeding complications	Vaginal bleeding	Subchorionic hemorrhage	Mucocutaneous hemorrhage	Postpartum hemorrhage
APOs	1.19 (0.64, 2.18) *p* = 0.586	0.69 (0.33, 1.46) *p* = 0.334	2.11 (0.80, 5.58) *p* = 0.132	3.39 (0.89, 12.97) *p* = 0.074	1.53 (0.47, 5.01) *p* = 0.485
Fetal death > 12 weeks	7.03 (0.77, 64.32) *p* = 0.084	2.69 (0.43, 16.72) *p* = 0.289	2.00 (0.21, 18.84) *p* = 0.544	10.73 (1.61, 71.74) *p* = 0.014	4.85 (0.47, 50.54) *p* = 0.186
Preterm delivery prior to 34 weeks	1.38 (0.52, 3.69) *p* = 0.522	0.46 (0.10, 2.08) *p* = 0.311	1.66 (0.44, 6.32) *p* = 0.458	8.30 (2.31, 29.84) *p* = 0.001	3.55 (0.86, 14.63) *p* = 0.079
Preeclampsia	0.42 (0.15, 1.19) *p* = 0.102	0.15 (0.02, 1.18) *p* = 0.071	0.32 (0.04, 2.52) *p* = 0.279	1.36 (0.28, 6.65) *p* = 0.703	2.38 (0.59, 9.55) *p* = 0.220
Placental Insufficiency	0.94 (0.49, 1.77) *p* = 0.839	0.63 (0.28, 1.41) *p* = 0.263	1.94 (0.76, 4.96) *p* = 0.165	1.88 (0.58, 6.09) *p* = 0.294	1.33 (0.40, 4.37) *p* = 0.640
SGA	0.71 (0.30, 1.67) *p* = 0.433	0.40 (0.11, 1.40) *p* = 0.151	0.88 (0.24, 3.23) *p* = 0.850	4.17 (1.22, 14.21) *p* = 0.023	1.84 (0.47, 7.27) *p* = 0.384
Preterm delivery < 37 weeks	1.36 (0.65, 2.83) *p* = 0.418	0.70 (0.27, 1.84) *p* = 0.473	2.26 (0.83, 6.16) *p* = 0.110	4.29 (1.30, 14.20) *p* = 0.017	1.31 (0.34, 5.12) *p* = 0.694
Fetal distress	0.50 (0.20, 1.26) *p* = 0.142	0.26 (0.06, 1.14) *p* = 0.074	1.38 (0.42, 4.47) *p* = 0.596	1.06 (0.22, 5.13) *p* = 0.941	NA
Fetal death	5.19 (0.53, 50.97) *p* = 0.157	4.06 (0.55, 29.86) *p* = 0.168	2.68 (0.27, 27.12) *p* = 0.402	4.88 (0.47, 50.86) *p* = 0.185	4.85(0.47, 50.54) *p* = 0.186

APS, antiphospholipid syndrome; APOs, adverse pregnancy outcomes; SGA, small for gestational age; NA, not applicable.

### Multivariate logistic regression analysis of the association of mucocutaneous hemorrhage with APOs

**Figure 1:**
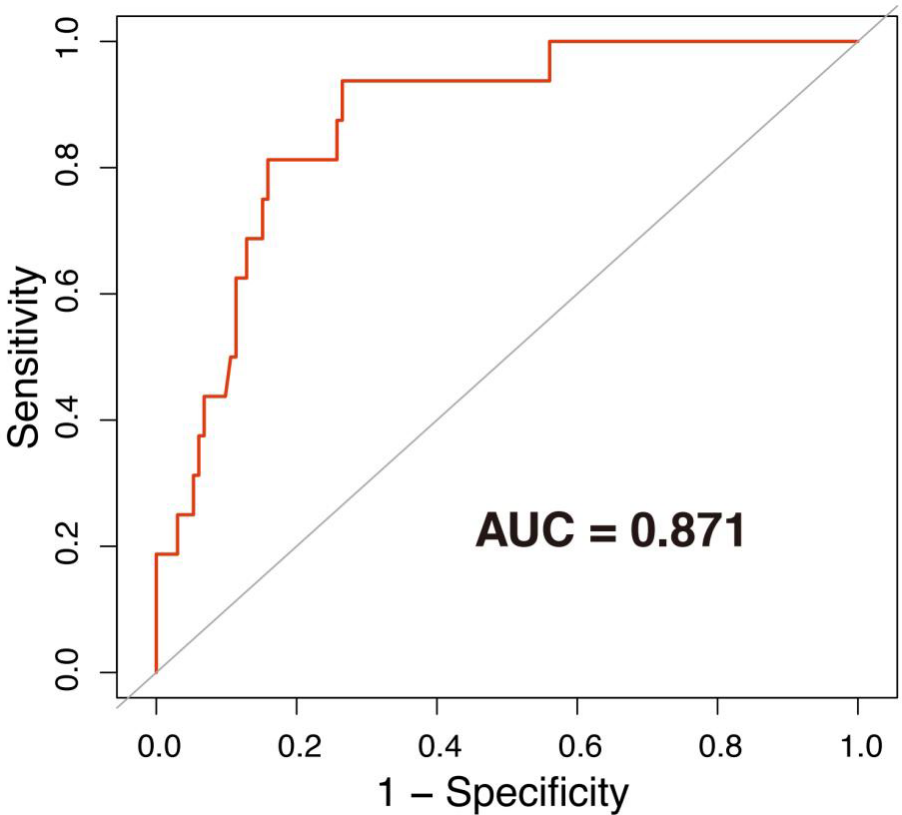
ROC curve of preterm delivery prior to 34 weeks. The area under the ROC curve (AUC) = 0.871. The associated factors used to draw the ROC were age, use of assisted reproduction techniques, APS before pregnancy, previous abortion history, thrombocytopenia, ANA positivity, high titer of aβ2-GPI/aCL, LDASA, LMWH, HCQ, Pred, and IVIG.

Mucocutaneous hemorrhage was significantly associated with fetal death after 12 weeks (OR = 37.77, 95% CI: 2.60–548.47, p = 0.008), preterm delivery prior to 34 weeks (OR = 18.25, 95% CI: 3.66–90.98, p = 0.001), and preterm delivery prior to 37 weeks (OR = 6.25, 95% CI: 1.62–24.09, p = 0.009) in model 1 adjusted for demographic confounders (**Table 3**). In the second model, which was adjusted for demographic and laboratory confounders, mucocutaneous hemorrhage was associated with preterm delivery prior to 34 weeks (OR = 35.30, 95% CI: 2.03–614.10, p = 0.015). After adjusting for the treatment method (i.e., LDASA, LMWH, HCQ, and Pred), mucocutaneous hemorrhage was still an independent risk factor for preterm delivery prior to 34 weeks (OR = 54.99, 95% CI: 2.04–1480.57, p = 0.0132). IVIG is applied in some severely ill patients. Five (83.33%) of the six patients had APOs (**Supplementary Table 3**). They did not have catastrophic APS. After adjustment for IVIG (model IV), we still found mucocutaneous hemorrhage as an indicator of preterm delivery prior to 34 weeks (OR = 40.29, 95% CI: 1.45–1121.32, p = 0.030). To further evaluate the accuracy of these factors for prediction of preterm delivery prior to 34 weeks, ROC analysis was conducted. The AUC was 0.871 (**Figure 1**). In hierarchical analysis, mucocutaneous hemorrhage was associated with preterm delivery prior to 34 weeks (OR = 13.56, 95% CI: 1.70–107.82, p = 0.01) and with fetal death after 20 weeks (OR = 43.33, 95% CI: 2.16–869.33, p = 0.014) in patients with LMWH (**Supplementary Table 4**).

**Table 3:** Multivariate logistic regression analyses of the association of mucocutaneous hemorrhage with APOs in patients with APS.

Exposure	Model I	Model II	Model III	Model IV
SGA	3.29 (0.83, 12.97) *p* = 0.089	3.96 (0.54, 29.08) *p* = 0.176	1.90 (0.21, 16.90) *p* = 0.566	3.69 (0.45, 30.42) *p* = 0.226
Fetal death > 12 weeks	37.77 (2.60, 548.47) *p* = 0.008	NA	NA	NA
Preterm delivery < 34 weeks	18.25 (3.66, 90.98) *p* < 0.001	35.30 (2.03, 614.10) *p* = 0.015	54.99 (2.04, 1480.57) *p* = 0.017	40.29 (1.45, 1121.32) *p* = 0.030
Preterm delivery < 37 weeks	6.25 (1.62, 24.09) *p* = 0.009	6.37 (0.93, 43.83) *p* = 0.060	5.35 (0.60, 47.41) *p* = 0.060	6.10 (0.72, 51.42) *p* = 0.096

Adjust I model adjusted for age, use of assisted reproduction techniques, APS before pregnancy, and previous abortion history.

Adjust II model adjusted for age, use of assisted reproduction techniques, APS before pregnancy, previous abortion history, thrombocytopenia, ANA positivity, and high titers of aβ2-GPIs/aCLs.

Adjust III model adjusted for age, use of assisted reproduction techniques, APS before pregnancy, previous abortion history, thrombocytopenia, ANA positivity, high titers of aβ2-GPIs/aCLs, LDASA, LMWH, HCQ, and Pred.

Adjust IV model adjusted for age, use of assisted reproduction techniques, APS before pregnancy, previous abortion history, thrombocytopenia, ANA positivity, high titers of aβ2-GPIs/aCLs, LDASA, LMWH, HCQ, Pred, and IVIG.

APS, antiphospholipid syndrome; APOs, adverse pregnancy outcomes; SGA, small for gestational age; ANA, antinuclear antibody; aβ2-GPIs, anti-β2 glycoprotein I antibodies; aCLs, anticardiolipin antibodies; LDASA, low-dose acetylsalicylic acid; LMWH, low–molecular weight heparin; HCQ, hydroxychloroquine; Pred, prednisone; IVIG, intravenous immunoglobulin; NA, not applicable.

### Risk factors of hemorrhage

When investigating the risk factors of hemorrhage, we observed that ANA positivity (OR = 3.78, 95% CI: 1.08–13.20, p = 0.04), persistent aβ2-GPIs/aCL positivity (OR = 13.11, 95% CI: 2.77–62.16, p = 0.0012), triple aPL positivity (OR = 6.29, 95% CI: 1.65–23.94, p = 0.0070), and double aPL positivity (OR = 3.91, 95% CI: 1.18–12.91, p = 0.0253) were associated with mucocutaneous hemorrhage (**Supplementary Table 5**).

## Discussion

In this retrospective study, we found that mucocutaneous hemorrhage was associated with APOs in patients with OAPS, whereas other bleeding events including vaginal bleeding, subchorionic hemorrhage, and postpartum hemorrhage were not. In addition, after adjusting for confounding factors such as demographics, laboratory indexes, and treatment, mucocutaneous hemorrhage was still found to independently influence the risk of preterm delivery prior to 34 weeks.

Considering that different antithrombotic therapies may affect the relationship between bleeding complications and APOs, we performed a hierarchical analysis and found that mucocutaneous hemorrhage was a significant predictor of preterm delivery prior to 34 weeks in the LMWH group. However, other treatments such as LDASA and LDASA combined with LMWH and other bleeding events were not found to be significantly associated with APOs. A previous study also showed no association between vaginal bleeding and preterm birth prior to 34 weeks (26). LDASA combined with LMWH is the main treatment for patients with OAPS, but whether these patients benefit from it has not been conclusively demonstrated. Most researchers assume that the combination of LDASA and prophylactic LMWH is safe because it is not associated with an increased risk of bleeding complications (24). However, some studies have shown that LMWH treatment does not significantly improve pregnancy outcomes for patients with OAPS (27). Although we found a significant association of mucocutaneous hemorrhage with APOs in the LMWH group in our study, the number of patients with mucocutaneous bleeding in this study was small, and the sample size of each treatment group became smaller after hierarchical analysis; therefore, a larger sample size is needed to demonstrate whether LMWH needs to be applied with caution in patients with OAPS.

We also found an increased risk of mucocutaneous hemorrhage in ANA-positive and triple aPL–positive patients, but not an increased risk of other bleeding events such as vaginal bleeding, subchorionic hemorrhage, or postpartum hemorrhage. Numerous studies have demonstrated an increased risk of APOs in triple aPL–positive patients, but it is not clear whether ANA positivity is associated with APOs (8, 9, 13–15). Some studies have found that ANA may negatively affect pregnancy outcomes, and the mechanism may be the deposition of ANA immune complexes in placental tissue, which activates the complement cascade and leads to tissue damage (28, 29). This may suggest a potential association between mucocutaneous hemorrhage and APOs in ANA-positive and triple aPL–positive patients with OAPS.

Few studies have reported the association between bleeding complications and APOs in patients with OAPS. This study reveals the risk factors and associations between mucocutaneous hemorrhage and APOs in patients with OAPS. When bleeding complications are observed in patients with OAPS, especially mucocutaneous hemorrhage, it is necessary to be aware of the occurrence of APOs. In addition, the choice of antithrombotic therapy for patients with OAPS with mucocutaneous hemorrhage may need to be emphasized.

Our study has several limitations. The retrospective design limits our ability to make definite conclusions about the association between APOs and bleeding complications. In addition, we used hierarchical analysis to determine the association of treatment with bleeding complications and APOs, but information may have been missing because of the small sample size in some hierarchies, and it could not determine whether the risk of APOs in the other treatment groups was increased due to the use of medications. Therefore, a larger sample size is needed to verify the conclusions of this study. Because of the lack of a control group and the usage of antithrombotic therapy, it is difficult to judge whether mucocutaneous hemorrhage is the cause of the APOs or just an outcome of active antithrombotic therapy.

## Conclusion

The study shows that mucocutaneous hemorrhage during pregnancy may be an indication of APOs in patients with OAPS. Whether LMWH should be used with caution in patients with OAPS with mucocutaneous hemorrhage and how to choose antithrombotic treatments for these patients need to be investigated on a larger sample of patients.

## Data availability statement

The original contributions presented in the study are included in the article/**Supplementary Material**. Further inquiries can be directed to the corresponding author.

## Ethics statement

The studies involving human participants were reviewed and approved by the ethics committee of Peking University People’s Hospital (2019PHB252). The patients/participants provided their written informed consent to participate in this study. Written informed consent was obtained from the individual(s) for the publication of any potentially identifiable images or data included in this article.

## Author contributions

YL, JJ, YY, and CL conceived of and designed the project. LH and YY collected and input the clinical and laboratory data. JJ completed the statistical analyses. JJ and YL conducted the calculations shown in the tables and figure. YL, JJ, YY, and CL wrote the manuscript. All authors provided critical feedback and helped design the research, perform analysis, and revise the manuscript. All authors contributed to the article and approved the submitted version.
