# Supplementary material for: Hemorrhage complications in obstetric antiphospholipid syndrome: Risk factors and association with adverse pregnancy outcomes
Source: Front Immunol. 2023 Mar 17;14:1145146. doi: 10.3389/fimmu.2023.1145146 (PMC10064047; doi:10.3389/fimmu.2023.1145146)
Supplement: Supplementary file 1 [file DataSheet_1.docx]

Supplementary Material

Hemorrhage complications in obstetric antiphospholipid syndrome：risk factors and association with adverse pregnancy outcomes

Yongjing Luo^1†^, Jiayang Jin^1,2†^, Yani Yan^3†^, Mengyao Zhang^1,2^, Lei Hou^4^，Yuke Hou^1,2^, Qiuyan Pei^3^，Chun Li^1,2*^

*** Correspondence:** Chun Li: 13811190098@163.com

# Supplementary Tables

Supplementary Table 1 Clinical and gestational characteristics between mucocutaneous hemorrhage and control group.

|  | Control group  (N=164) | Mucocutaneous hemorrhage group  (N=12) | *P* |
| --- | --- | --- | --- |
| Age | 33.44 ± 3.56 | 29.92 ± 3.96 | 0.002 |
| Onset-age | 31.42 ± 3.56 | 28.58 ± 4.21 | 0.005 |
| Primipara, n (%) | 122 (74.39%) | 8 (66.67%) | 0.557 |
| Diagnosed APS before pregnancy, n (%) | 123 (75.00%) | 5 (41.67%) | 0.012 |
| Gestational week | 37.49 ± 3.11 | 32.00 ± 9.43 | 0.004 |
| Previous abortion history, n (%) | 117 (71.34%) | 5 (41.67%) | 0.031 |
| Use of assisted reproduction techniques, n (%) | 41 (25.00%) | 0 (0.00%) | 0.048 |
| Cesarean section, n (%) | 93 (56.71%) | 8 (66.67%) | 0.501 |
| Treatment, n (%) | 162 (98.78%) | 12 (100.00%) | 0.999 |
| Pred, mg/d | 0.00 (0.00-5.00) | 10.00(3.75-15.00) | <0.001 |
| Pred, n (%) | 46 (28.05%) | 9 (75.00%) | <0.001 |
| HCQ, mg/d | 400 (0.00-400.00) | 400 (200.00-400.00) | 0.541 |
| HCQ, n (%) | 118 (71.95%) | 10 (83.33%) | 0.393 |
| LMWH, IU/d | 4100(4000.00-5000.00) | 0.00(0.00-4000.00) | <0.001 |
| LMWH, n (%) | 134 (81.71%) | 4 (33.33%) | <0.001 |
| LDASA, mg/d | 75.00(75.00-100.00) | 0.00(0.00-0.00) | <0.001 |
| LDASA, n (%) | 122(74.39%) | 0(0.00%) | <0.001 |
| LMWH + LDASA, n (%) | 98 (59.76%) | 0 (0.00%) | <0.001 |
| IVIG, n (%) | 4 (2.44%) | 2 (16.67%) | 0.055 |
| Thrombus, n (%) | 3 (1.83%) | 0 (0.00%) | 0.999 |
| Thrombocytopenia, n (%) | 23 (14.02%) | 12 (100.00%) | <0.001 |
| ANA positivity, n (%) | 33 (24.09%) | 6 (54.55%) | 0.027 |
| Hypocomplementemia, n (%) | 20 (13.51%) | 3 (27.27%) | 0.211 |
| High titers of aβ2-GPIs/aCLs, n (%) | 45 (27.61%) | 10 (83.33%) | <0.001 |
| Three aPLs positivity, n (%) | 12 (7.36%) | 4 (33.33%) | 0.003 |
| Two aPLs positivity, n (%) | 34 (20.86%) | 6 (50.00%) | 0.020 |
| APOs, n (%) | 77 (46.95%) | 9 (75.00%) | 0.061 |
| Fetal death after 12 weeks of gestation, n (%) | 3 (1.83%) | 2 (16.67%) | 0.038 |
| Preterm delivery before 34 weeks, n (%) | 13 (7.93%) | 5 (41.67%) | <0.001 |
| Preeclampsia, n (%) | 21 (12.80%) | 2 (16.67%) | 0.702 |
| Placental Insufficiency, n (%) | 57 (34.76%) | 6 (50.00%) | 0.288 |
| SGA, n (%) | 24 (14.63%) | 5 (41.67%) | 0.015 |
| Fetal death, n (%) | 3 (1.83%) | 1 (8.33%) | 0.248 |
| Preterm delivery <37 weeks, n (%) | 31(18.90%) | 6(50.00%) | 0.011 |
| Oligoamnios, n (%) | 34(20.73%) | 1(8.33%) | 0.299 |
| Fetal distress, n (%) | 26(15.85%) | 2(16.67%) | 0.941 |
| Vaginal bleeding, n (%) | 36 (21.95%) | 0 (0.00%) | 0.069 |
| Subchorionic hemorrhage, n (%) | 19 (11.59%) | 1 (8.33%) | 0.732 |
| Postpartum hemorrhage, ml | 327.13 ± 247.75 | 581.82 ± 316.44 | 0.004 |
| Abnormal umbilical blood flow, n (%) | 6 (3.66%) | 0 (0.00%) | 0.999 |
| Abnormal middle cerebral artery blood flow, n (%) | 3 (1.83%) | 0 (0.00%) | 0.999 |

APS, antiphospholipid syndrome; Pred, prednisone; HCQ, hydroxychloroquine; LMWH, low molecular weight heparin; LDASA, low dose acetylsalicylic acid; IVIG, intravenous immunoglobulin; ANA, antinuclear antibody; aβ2-GPI, anti-β2 glycoprotein I antibodies; aCL, anticardiolipin antibodies; aPLs, antiphospholipid antibodies. APOs, adverse pregnancy outcomes; SGA, small for gestational age.

Supplementary Table 2 Hemorrhagic complications in the presence or absence of antithrombotics.

|  | Without antithrombotics  n = 15 | With antithrombotics  n = 161 | p value |
| --- | --- | --- | --- |
| Bleeding complications, n (%) | 12 (80.00%) | 54 (33.54%) | <0.001 |
| Subchorionic hemorrhage, n (%) | 1 (6.67%) | 19 (11.80%) | 0.549 |
| Mucocutaneous hemorrhage, n (%) | 9 (60.00%) | 3 (1.91%) | <0.001 |
| Vaginal bleeding, n (%) | 2 (13.33%) | 32 (20.95%) | 0.513 |
| Postpartum hemorrhage, n (%) | 3 (21.43%) | 9 (5.59%) | 0.025 |

Supplementary Table 4 Hierarchical analysis

| Bleeding complications |  |  |  |  |  |  |  |  |
| --- | --- | --- | --- | --- | --- | --- | --- | --- |
|  | APOs | Preterm delivery prior to 34 weeks | Preeclampsia | Placental Insufficiency | SGA | Preterm delivery prior to 37 weeks | Fetal distress | Fetal death ≥ 20 weeks |
| Antithrombotic therapy |  |  |  |  |  |  |  |  |
| No | 4.00 (0.27, 58.56)  p = 0.3113 | NA | NA | 2.00 (0.14, 28.42)  p = 0.609 | 1.43 (0.10, 20.44)  p = 0.793 | NA | 0.40(0.02, 6.85)  p = 0.527 | NA |
| Yes | 1.10 (0.56, 2.15)  p = 0.785 | 0.62 (0.16, 2.36)  p = 0.482 | 0.21 (0.05, 0.93)  p = 0.039 | 0.90 (0.44, 1.82)  p = 0.765 | 0.40(0.13, 1.25)  p = 0.117 | 1.02 (0.44, 2.37)  p = 0.956 | 0.48 (0.17, 1.37)  p = 0.172 | 4.42(0.39, 49.90)  p = 0.230 |
| LMWH |  |  |  |  |  |  |  |  |
| No | 2.40 (0.61, 9.38)  p = 0.2079 | 2.30 (0.43, 12.25)  p = 0.3278 | 0.71 (0.06, 8.67)  p = 0.7916 | 2.17 (0.57, 8.26)  p = 0.257 | 2.25 (0.49, 10.34)  p = 0.297 | 2.25 (0.49, 10.34)  p = 0.297 | 0.85 (0.17, 4.26)  p = 0.8432 | 1.50 (0.09, 26.01)  p = 0.781 |
| Yes | 1.05 (0.52, 2.13)  p = 0.894 | 1.08 (0.30, 3.88)  p = 0.9104 | NA | 0.78 (0.36, 1.67)  p = 0.523 | 0.42 (0.13, 1.33)  p = 0.141 | 1.28 (0.54, 3.01)  p = 0.578 | 0.42 (0.13, 1.33)  p = 0.141 | NA |
| LDASA |  |  |  |  |  |  |  |  |
| No | 1.21 (0.41, 3.63)  p = 0.728 | 3.14 (0.57, 17.23)  p = 0.189 | 0.40 (0.07, 2.42)  p = 0.321 | 1.27 (0.43, 3.76)  p = 0.662 | 1.03 (0.31, 3.41)  p = 0.963 | 1.66 (0.52, 5.29)  p = 0.388 | 0.53 (0.13, 2.14)  p = 0.371 | NA |
| Yes | 1.10 (0.50, 2.44)  p = 0.816 | 0.60 (0.12, 2.99)  p = 0.534 | 0.15 (0.02, 1.22)  p = 0.076 | 0.70 (0.29, 1.68)  p = 0.420 | 0.17 (0.02, 1.34)  p = 0.092 | 0.87 (0.29, 2.65)  p = 0.812 | 0.45 (0.12, 1.67)  p = 0.234 | 2.55 (0.15, 41.90)  p = 0.513 |
| LWMH + LDASA |  |  |  |  |  |  |  |  |
| No | 1.52 (0.60, 3.85)  p = 0.3755 | 1.80 (0.50, 6.52)  p = 0.3705 | 0.42 (0.08, 2.24)  p = 0.3117 | 1.49 (0.60, 3.72)  p = 0.3922 | 1.13 (0.39, 3.29)  p = 0.8181 | 1.57 (0.58, 4.27)  p = 0.3752 | 0.63 (0.19, 2.06)  p = 0.4447 | 2.87 (0.25, 33.06)  p = 0.3986 |
| Yes | 0.98 (0.41, 2.33)  p = 0.9558 | 0.87 (0.16, 4.77)  p = 0.8739 | 0.16 (0.02, 1.25)  p = 0.0805 | 0.54 (0.19, 1.51)  p = 0.2366 | 0.19 (0.02, 1.58)  p = 0.1255 | 1.12 (0.35, 3.62)  p = 0.8497 | 0.36 (0.07, 1.72)  p = 0.1998 | NA |

| Vaginal bleeding |  |  |  |  |  | |  |  |  |  |
| --- | --- | --- | --- | --- | --- | --- | --- | --- | --- | --- |
|  | APOs | Fetal death (>12 weeks) | Preterm delivery prior to 34 weeks | Preeclampsia | Placental Insufficiency | SGA | | Preterm delivery prior to 37 weeks | Fetal distress | Fetal death ≥ 20 weeks |
| Antithrombotic therapy |  |  |  |  |  |  | |  |  |  |
| No | 0.63 (0.03, 12.41)  p = 0.7579 | 5.50 (0.23, 128.97)  p = 0.2896 | 2.25 (0.11, 45.73)  p = 0.5977 | NA | 1.17 (0.06, 22.94)  p = 0.9192 | 1.60 (0.08, 31.77)  p = 0.7579 | | 1.60 (0.08, 31.77)  p = 0.7579 | NA | NA |
| Yes | 0.67 (0.31, 1.48)  p = 0.3251 | 4.00 (0.24, 65.75)  p = 0.3318 | 0.30 (0.04, 2.43)  p = 0.2612 | NA | 0.65 (0.28, 1.52)  p = 0.3203 | 0.33 (0.07, 1.49)  p = 0.1493 | | 0.71 (0.25, 2.01) p = 0.5133 | 0.30 (0.07, 1.33)  p = 0.1116 | 1.98 (0.17, 22.59)  p = 0.5810 |
| LMWH |  |  |  |  |  |  | |  |  |  |
| No | 1.32 (0.19, 9.02)  p = 0.7747 | 2.42 (0.20, 29.24)  p = 0.4878 | 1.08 (0.10, 11.52)  p = 0.9471 | NA | 1.70 (0.25, 11.59)  p = 0.5879 | 0.75 (0.07, 7.73)  p = 0.809 | | 0.75 (0.07, 7.73) p = 0.8090 | NA | 7.75 (0.40, 149.71)  p = 0.1753 |
| Yes | 0.59 (0.25, 1.36)  p = 0.2167 | NA | 0.34 (0.04, 2.80)  p = 0.3174 | NA | 0.57 (0.22, 1.46)  p = 0.2396 | 0.36 (0.08, 1.66)  p = 0.1912 | | 0.75 (0.26, 2.19) p = 0.6008 | 0.36 (0.08, 1.66)  p = 0.1912 | 3.75 (0.23, 61.85)  p = 0.3554 |
| LDASA |  |  |  |  |  |  | |  |  |  |
| No | 0.50 (0.12, 2.14)  p = 0.3533 | 2.62 (0.21, 32.52)  p = 0.4523 | 0.66 (0.07, 6.15)  p = 0.7157 | NA | 0.88 (0.21, 3.70)  p = 0.8574 | 0.68 (0.12, 3.73) p = 0.6581 | | 0.97 (0.21, 4.42) p = 0.9651 | 0.00 (0.00, Inf)  p = 0.9936 | 5.37 (0.30, 95.06)  p = 0.2512 |
| Yes | 0.76 (0.31, 1.86)  p = 0.5440 | 3.87 (0.23, 64.23)  p = 0.3444 | 0.39 (0.05, 3.26)  p = 0.3873 | NA | 0.61 (0.22, 1.68)  p = 0.3414 | 0.26 (0.03, 2.09)  p = 0.2047 | | 0.66 (0.18, 2.49)  p = 0.5446 | 0.42 (0.09, 1.98)  p = 0.2753 | 3.87 (0.23, 64.23)  p = 0.3444 |
| LMWH+ LDASA |  |  |  |  |  |  | |  |  |  |
| No | 0.73 (0.21, 2.51)  p = 0.6172 | 1.85 (0.18, 19.43)  p = 0.6087 | 0.49 (0.06, 4.24)  p = 0.5177 | NA | 1.13 (0.33, 3.89)  p = 0.8424 | 0.60 (0.12, 3.03)  p = 0.5366 | | 0.79 (0.19, 3.24)  p = 0.7429 | 0.00 (0.00, Inf)  p = 0.9926 | 2.82 (0.23, 33.81)  p = 0.4138 |
| Yes | 0.67 (0.25, 1.79)  p = 0.4279 | inf. (0.00, Inf)  p = 0.9972 | 0.54 (0.06, 4.74)  p = 0.5779 | NA | 0.49 (0.15, 1.62)  p = 0.2437 | 0.30 (0.04, 2.52)  p = 0.2705 | | 0.82 (0.21, 3.20)  p = 0.7701 | 0.57 (0.12, 2.80)  p = 0.4916 | inf. (0.00, Inf)  p = 0.9972 |

| Mucocutaneous hemorrhage |  |  |  |  |  |  |  |  |  |
| --- | --- | --- | --- | --- | --- | --- | --- | --- | --- |
|  | APOs | Fetal death after 12 weeks of gestation | Preterm delivery prior to 34 weeks | Preeclampsia | Placental Insufficiency | SGA | Preterm delivery prior to 37 weeks | Fetal distress | Fetal death ≥ 20 weeks |
| Antithrombotic therapy |  |  |  |  |  |  |  |  |  |
| No | 7.00 (0.69, 70.75)  p = 0.099 | 1.43 (0.10, 20.44)  p = 0.793 | 4.00 (0.32, 49.60)  p = 0.281 | NA | 2.50 (0.29, 21.40)  p = 0.403 | 1.60 (0.19, 13.70)  p = 0.668 | 6.25 (0.50, 77.49)  p = 0.154 | 1.43 (0.10, 20.44)  p = 0.793 | NA |
| Yes | 2.16 (0.19, 24.35)  p = 0.533 | NA | 5.92 (0.50, 70.07)  p = 0.159 | 3.55 (0.31, 41.09)  p = 0.310 | 0.90 (0.08, 10.15)  p = 0.932 | 3.00 (0.26, 34.51)  p = 0.378 | 2.07 (0.18, 23.55)  p = 0.559 | NA | 38.00 (2.37, 610.33)  p = 0.0102 |
| LMWH |  |  |  |  |  |  |  |  |  |
| No | 3.21 (0.55, 18.65)  p = 0.1931 | 4.50 (0.52, 38.65)  p = 0.1704 | 3.75 (0.63, 22.20)  p = 0.1452 | 1.93 (0.15, 24.46)  p = 0.6123 | 2.05 (0.41, 10.24)  p = 0.3811 | 4.80 (0.89, 25.96)  p = 0.0685 | 4.80 (0.89, 25.96)  p = 0.0685 | 1.28 (0.20, 8.01)  p = 0.7935 | NA |
| Yes | 3.34 (0.34, 32.94)  p = 0.3019 | NA | 13.56 (1.70, 107.82)  p = 0.0137 | 2.24 (0.22, 22.74)  p = 0.4968 | 0.66 (0.07, 6.52)  p = 0.7215 | 1.96 (0.19, 19.89)  p = 0.5674 | 4.04 (0.54, 30.03)  p = 0.1727 | NA | 43.33 (2.16, 869.33) 0.0138 |
| LDASA |  |  |  |  |  |  |  |  |  |
| No | 2.59 (0.61, 10.98)  p = 0.1962 | 8.00 (0.66, 97.31)  p = 0.1028 | 9.05 (1.75, 46.77)  p = 0.0086 | 1.85 (0.30, 11.60)  p = 0.5112 | 1.16 (0.32, 4.20)  p = 0.8234 | 2.21 (0.57, 8.55)  p = 0.2488 | 2.42 (0.65, 9.01)  p = 0.1889 | 0.82 (0.15, 4.53)  p = 0.8248 | 3.64 (0.21, 62.93)  p = 0.3748 |
| Yes | NA | NA | NA | NA | NA | NA | NA | NA | NA |
| LMWH + LDASA |  |  |  |  |  |  |  |  |  |
| No | 2.65 (0.66, 10.69)  p = 0.1717 | 6.20 (0.78, 49.17)  p = 0.0842 | 6.90 (1.66, 28.64)  p = 0.0078 | 1.93 (0.34, 10.96)  p = 0.4565 | 1.13 (0.33, 3.89)  p = 0.8424 | 2.80 (0.76, 10.27)  p = 0.1201 | 3.00 (0.85, 10.63)  p = 0.0888 | 0.78 (0.15, 4.03)  p = 0.7713 | 2.82 (0.23, 33.81)  p = 0.4138 |
| Yes | NA | NA | NA | NA | NA | NA | NA | NA | NA |

| Postpartum hemorrhage |  |  |  |  |  |  |  |  |  |
| --- | --- | --- | --- | --- | --- | --- | --- | --- | --- |
|  | APOs | Fetal death after 12 weeks of gestation | Preterm delivery prior to 34 weeks | Preeclampsia | Placental Insufficiency | SGA | Preterm delivery prior to 37 weeks | Fetal distress | Fetal death ≥ 20 weeks |
| LMWH |  |  |  |  |  |  |  |  |  |
| No | 3.00 (0.28, 31.99)  p = 0.363 | 5.00 (0.34, 72.77)  p = 0.239 | 7.00 (0.76, 64.61)  p = 0.086 | 5.00 (0.34, 72.77)  p = 0.239 | 3.86 (0.36, 41.20)  p = 0.264 | 4.33 (0.50, 37.26)  p = 0.182 | 4.33 (0.50, 37.26)  p = 0.182 | NA | NA |
| Yes | 1.46 (0.32, 6.81)  p = 0.627 | NA | 1.97 (0.22, 17.99)  p = 0.549 | 1.09 (0.12, 9.60)  p = 0.939 | 0.79 (0.15, 4.24)  p = 0.784 | 0.96 (0.11, 8.39)  p = 0.968 | 0.62 (0.07, 5.40)  p = 0.668 | NA | 21.17 (1.18, 380.94)  p = 0.039 |
| LDASA |  |  |  |  |  |  |  |  |  |
| No | 2.33 (0.23, 24.08)  p = 0.4767 | 15.67 (0.77, 317.05)  p = 0.073 | 33.00 (2.75, 395.61)  p = 0.006 | 11.00 (1.21, 100.39)  p = 0.034 | 3.86 (0.37, 39.80)  p = 0.2569 | 10.09 (0.95, 107.00)  p = 0.055 | 7.29 (0.70, 76.18)  p = 0.097 | NA | 15.67 (0.77, 317.05)  p = 0.073 |
| Yes | 1.65 (0.35, 7.73)  p = 0.5229 | NA | NA | NA | 0.84 (0.16, 4.56)  p = 0.8443 | NA | NA | NA | NA |
| LWMH + LDASA |  |  |  |  |  |  |  |  |  |
| No | 3.37 (0.36, 31.68)  p = 0.288 | 8.50 (0.63, 114.87)  p = 0.107 | 13.50 (1.92, 95.09)  p = 0.009 | 7.11 (0.99, 51.26)  p = 0.0516 | 5.03 (0.53, 47.34)  p = 0.1577 | 6.00 (0.91, 39.42)  p = 0.0621 | 4.33 (0.67, 28.05)  p = 0.124 | NA | 8.50 (0.63, 114.87)  p = 0.107 |
| Yes | 1.31 (0.25, 6.83)  p = 0.751 | NA | NA | NA | 0.49 (0.05, 4.42)  p = 0.527 | NA | NA | NA | NA |
| Antithrombotic therapy |  |  |  |  |  |  |  |  |  |
| No | 1.67 (0.11, 24.26)  p = 0.709 | 5.00 (0.21, 117.90)  p = 0.3182 | 9.00 (0.52, 155.25)  p = 0.1305 | NA | 3.50 (0.24, 51.90)  p = 0.3625 | 5.33 (0.34, 82.83)  p = 0.2316 | 5.33 (0.34, 82.83)  p = 0.2316 | NA | NA |
| Yes | 1.83 (0.42, 7.94)  p = 0.419 | NA | 1.63 (0.18, 14.38)  p = 0.6596 | 0.98 (0.11, 8.39)  p = 0.9834 | 1.09 (0.25, 4.73)  p = 0.9116 | 0.82 (0.10, 7.03)  p = 0.8601 | 0.57 (0.07, 4.78)  p = 0.6018 | NA | 10.50 (0.85, 130.16)  p = 0.0671 |

| Subchorionic hemorrhage |  |  |  |  |  |  |  |  |  |
| --- | --- | --- | --- | --- | --- | --- | --- | --- | --- |
|  | APOs | Fetal death after 12 weeks of gestation | Preterm delivery prior to 34 weeks | Preeclampsia | Placental Insufficiency | SGA | Preterm delivery prior to 37 weeks | Fetal distress | Dead fetus ≥ 20 weeks |
| Antithrombotic therapy |  |  |  |  |  |  |  |  |  |
| No | NA | NA | NA | NA | NA | NA | NA | NA | NA |
| yes | 2.34 (0.83, 6.60)  p = 0.1068 | 8.12 (0.49, 135.80)  p = 0.1451 | 1.45 (0.30, 7.16)  p = 0.6449 | 0.37 (0.05, 2.96)  p = 0.3494 | 2.53 (0.93, 6.83)  p = 0.0677 | 1.19 (0.32, 4.49)  p = 0.7972 | 2.28 (0.78, 6.66)  p = 0.1316 | 1.61 (0.48, 5.35)  p = 0.4410 | 4.03 (0.35, 46.83)  p = 0.2654 |
| LMWH |  |  |  |  |  |  |  |  |  |
| No | 1.78 (0.15, 21.51)  p = 0.6510 | NA | NA | NA | 2.25 (0.19, 27.22)  p = 0.5238 | NA | NA | 1.93 (0.15, 24.46)  p = 0.6123 | NA |
| Yes | 2.65 (0.87, 8.09)  p = 0.0875 | NA | 3.20 (0.75, 13.60)  p = 0.1147 | 0.40 (0.05, 3.23)  p = 0.3898 | 2.22 (0.77, 6.36)  p = 0.1389 | 1.38 (0.36, 5.38)  p = 0.6382 | 3.63 (1.21, 10.84)  p = 0.0210 | 1.38 (0.36, 5.38)  p = 0.6382 | 7.87 (0.47, 132.43)  p = 0.1522 |
| LDASA |  |  |  |  |  |  |  |  |  |
| No | 2.40 (0.44, 13.20)  p = 0.3142 | NA | 0.78 (0.08, 7.32)  p = 0.8243 | NA | 2.08 (0.44, 9.79)  p = 0.3526 | 0.82 (0.15, 4.61)  p = 0.8222 | 2.21 (0.48, 10.15)  p = 0.3063 | 1.54 (0.26, 9.08)  p = 0.6324 | NA |
| Yes | 2.27 (0.63, 8.22)  p = 0.211 | 10.70 (0.62, 184.35)  p = 0.103 | 2.78 (0.51, 15.10)  p = 0.237 | 0.67 (0.08, 5.65)  p = 0.714 | 1.89 (0.54, 6.65)  p = 0.319 | 0.73 (0.09, 6.18)  p = 0.773 | 2.16 (0.52, 9.00)  p = 0.292 | 1.28 (0.25, 6.47)  p = 0.767 | 10.70 (0.62, 184.35)  p = 0.103 |
| LWMH + LDASA |  |  |  |  |  |  |  |  |  |
| No | 2.29 (0.56, 9.40)  p = 0.2517 | NA | 0.55 (0.06, 4.78)  p = 0.5880 | NA | 2.17 (0.58, 8.15)  p = 0.2501 | 0.68 (0.13, 3.48)  p = 0.6441 | 1.49 (0.39, 5.72)  p = 0.5593 | 1.66 (0.38, 7.18)  p = 0.5004 | NA |
| Yes | 2.30 (0.52, 10.22)  p = 0.275 | NA | 5.53 (0.88, 34.75)  p = 0.068 | 0.90 (0.10, 8.02)  p = 0.928 | 1.60 (0.35, 7.22)  p = 0.5408 | 1.11 (0.12, 10.02)  p = 0.9231 | 3.80 (0.80, 18.00)  p = 0.093 | 0.90 (0.10, 8.02)  p = 0.9284 | NA |

APS, antiphospholipid syndrome; APOs, adverse pregnancy outcomes; SGA, small for gestational age; LMWH, low-molecular-weight heparin; LDASA, low dose acetylsalicylic acid; NA, Not Applicable.

Supplementary Table 5 Risk factors for hemorrhage

|  | Mucocutaneous hemorrhage | Bleeding complications | Vaginal bleeding | Subchorionic hemorrhage | Postpartum hemorrhage |
| --- | --- | --- | --- | --- | --- |
| Thrombocytopenia | NA | 2.75 (1.29, 5.87)  p = 0.0087 | 0.31 (0.09, 1.07)  p = 0.0631 | 1.40 (0.47, 4.15)  p = 0.5443 | 2.22 (0.63, 7.85)  p = 0.2171 |
| ANA positivity | 3.78 (1.08, 13.20)  p = 0.0370 | 1.14 (0.54, 2.39)  p = 0.7257 | 0.87 (0.35, 2.13)  p = 0.7552 | 1.34 (0.47, 3.82)  p = 0.5805 | 0.23 (0.03, 1.88)  p = 0.1719 |
| Hypocomplementemia | 2.40 (0.59, 9.81)  p = 0.2230 | 0.81 (0.32, 2.04)  p = 0.6548 | 0.14 (0.02, 1.05)  p = 0.0557 | 1.83 (0.55, 6.16)  p = 0.3263 | 0.54 (0.07, 4.41)  p = 0.5663 |
| Persistent aβ2GPIs/ aCLs | 13.11 (2.77, 62.16)  p = 0.0012 | 1.44 (0.75, 2.76)  p = 0.2748 | 0.67 (0.29, 1.55)  p = 0.3531 | 0.70 (0.24, 2.03)  p = 0.5122 | 2.31 (0.71, 7.51)  p = 0.1652 |
| Triple aPLs positivity | 6.29 (1.65, 23.94)  p = 0.0070 | 1.32 (0.47, 3.73)  p = 0.6021 | NA | 1.93 (0.50, 7.45)  p = 0.3415 | 2.11 (0.42, 10.62)  p = 0.3633 |
| Double aPLs positivity | 3.91 (1.18, 12.91)  p = 0.0253 | 1.22 (0.59, 2.53)  p = 0.5910 | 0.84 (0.33, 2.09)  p = 0.7019 | 1.18 (0.40, 3.47)  p = 0.7683 | 0.29 (0.04, 2.35)  p = 0.2488 |
| LAC positivity | 2.33 (0.71, 7.57)  p = 0.1608 | 1.15 (0.60, 2.22)  p = 0.6729 | 0.80 (0.36, 1.81)  p = 0.5969 | 0.93 (0.34, 2.56)  p = 0.8838 | 1.60 (0.48, 5.29)  p = 0.4408 |

APS, antiphospholipid syndrome; APOs, adverse pregnancy outcomes; SGA, small for gestational age; ANA, antinuclear antibody; LAC, lupsu anticoagulant; NA, Not Applicable.
